# Reference values for shear wave elastography, shear wave dispersion and attenuation imaging in healthy paediatric livers

**DOI:** 10.1007/s00247-025-06434-9

**Published:** 2025-11-01

**Authors:** Michael Zellner, Magdalena Schmidt, Florian Huber, Catherine Mary Paverd, Alexander Martin, Srdjan Micic, André Eichenberger, Vasiliki Spyropoulou, Karla Drommelschmidt, Christian J. Kellenberger

**Affiliations:** 1 Department of Diagnostic Imaging, University Children’s Hospital Zurich, Lenggstrasse 30, Zurich, 8008 Switzerland; 2 Diagnostic and Interventional Radiology, University Hospital of Zurich, Zurich, Switzerland; 3 Department of Gastroenterology and Hepatology, University Children’s Hospital Zurich, Zurich, Switzerland; 4 University of Tübingen, Tübingen, Germany

**Keywords:** Child, Liver, Liver diseases, Reference values, Ultrasonography

## Abstract

**Background:**

The rising prevalence of paediatric liver disease, including metabolic dysfunction- associated steatotic liver disease, highlights the need for reliable, non-invasive diagnostic tools. Advanced ultrasound techniques such as shear wave elastography (SWE), shear wave dispersion (SWD), and attenuation imaging (ATI) offer promising alternatives to biopsy or magnetic resonance imaging, but normative paediatric values remain limited.

**Objective:**

This study aimed to establish age-specific reference values for SWE, SWD, and ATI in healthy children and to assess potential influencing factors such as age, sex, body mass index (BMI), and fasting duration.

**Materials and methods:**

In this retrospective study, 264 children (135 female, median age 11.5 years) without known liver disease were selected from a cohort of 734. Each child underwent liver ultrasound using a standardized protocol with five ATI and ten SWE/SWD measurements. Only high-quality data were included. Statistical analyses examined correlations between imaging parameters and patient characteristics.

**Results:**

The median ATI was 0.54 dB/cm/MHz [interquartile range (IQR):0.50–0.58], SWE was 1.24 m/s (IQR:1.14–1.33) and SWD was 11.70 (m/s)/kHz (IQR:10.84–12.13). ATI and SWD values showed significant negative correlations with age (*P* < 0.001 and *P* = 0.0048, respectively). SWD also correlated negatively with BMI *z*-score (*P* < 0.001) and was significantly lower in females (*P* = 0.001). SWE showed only a weak positive correlation with measurement depth (*P* = 0.0261). Fasting duration had no significant impact on any measurement.

**Conclusion:**

This study provides reference values for SWE, SWD, and ATI in children. Age and sex influence SWD and ATI values, underscoring the importance of age-specific interpretation in paediatric liver ultrasound.

**Graphical Abstract:**

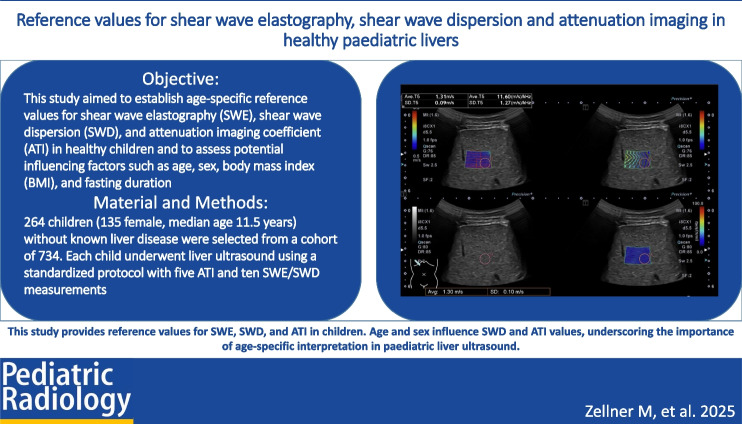

**Supplementary information:**

The online version contains supplementary material available at 10.1007/s00247-025-06434-9.

## Introduction

The incidence of both acute and chronic liver diseases in children is on the rise, with metabolic dysfunction-associated steatotic liver disease now recognized as the most common cause of paediatric liver pathology [1]. The prevalence of metabolic dysfunction- associated steatotic liver disease in children is now approaching the levels observed in adults, affecting 7.6% of the general paediatric population and over 30% of children with obesity [2]. Metabolic dysfunction-associated steatotic liver disease contributes not only to liver-specific morbidity but is also linked to a higher risk of metabolic syndrome, including type 2 diabetes, cardiovascular disease, and increased mortality in adulthood, imposing a significant economic burden [2, 3]. Because of the dramatic rise in global metabolic risk factors and an aging population, the burden of advanced disease from metabolic dysfunction-associated steatotic liver disease is projected to more than double between 2016 and 2030. This underscores the critical need for early detection, particularly in paediatric patients, to prevent progression to advanced liver disease later in life. Especially, metabolic risk factors in children and adolescents represent one of the greatest threats to global health in the coming decades [4]. Unlike obesity-related liver conditions, which are more prevalent in industrialized nations and progress with age, genetic disorders such as ciliopathies, Alagille syndrome, alpha-1 antitrypsin deficiency, and various genetic cholestatic liver diseases often involve liver complications starting in early childhood [1]. These conditions underscore the critical need for precise and sensitive screening methods to detect subtle liver tissue changes early, enabling timely diagnosis and, where possible, the initiation of treatment [3].

The imaging assessment of liver tissue in children currently relies predominantly on ultrasonography, a well-established, non-invasive imaging technique that is particularly effective in paediatric patients due to their favourable body composition, allowing for high-resolution imaging [5, 6]. However, conventional ultrasound is limited in its ability to provide detailed information beyond organ structure, size, and perfusion. Accurate evaluation and quantitative staging of liver fibrosis and steatosis still necessitate invasive liver biopsy or magnetic resonance imaging, often requiring general anaesthesia in younger children. Recent advancements in ultrasound technology, such as shear wave elastography, shear wave dispersion, and attenuation imaging, offer enhanced imaging capabilities and present a promising alternative to magnetic resonance imaging and computed tomography scans for more detailed liver tissue analysis.

The attenuation imaging technique enables the quantification of the attenuation coefficient, which reflects the variation in ultrasound intensity as it propagates through liver tissue [7]. Attenuation imaging demonstrates improved precision and lower variability compared to the controlled attenuation parameter used in FibroScan® systems and surpasses grayscale grading on B-mode ultrasound, providing a more precise and quantitative assessment of liver tissue attenuation (steatosis) in the adult population [8, 9].

Shear wave elastography measures the speed of shear waves generated by focused ultrasound push pulses, which cause lateral displacement of tissue. Among the emerging quantitative ultrasound techniques, shear wave elastography is the most widely established and provides a reliable assessment of tissue elasticity. Its clinical utility has been well-documented, demonstrating effectiveness in detecting and staging liver fibrosis [10, 11]. Furthermore, shear wave elastography shows great promise as an advanced diagnostic tool for the early detection and severity assessment of veno-occlusive disease, potentially improving clinical outcomes in paediatric patients [12].

Shear wave dispersion imaging evaluates the shear wave slope, providing critical insights into tissue viscosity linked to oedema, necrosis, allograft damage, and cellular inflammation. This innovative technique offers a more detailed assessment of tissue properties than conventional ultrasound methods [13–15].

This study aimed to establish normal reference values for the non-invasive techniques of shear wave elastography, shear wave dispersion, and attenuation imaging in healthy children while considering potential influencing factors. This study therefore provides additional useful information to build on the existing body of literature, which is important given that studies to date have only investigated shear wave elastography, shear wave dispersion, or attenuation imaging individually rather than in combination, have lacked sufficient paediatric-specific data, have not accounted for key confounding variables, or have only used limited patient numbers [6, 16–19].

## Material and methods

### Patients

**Fig. 1:**
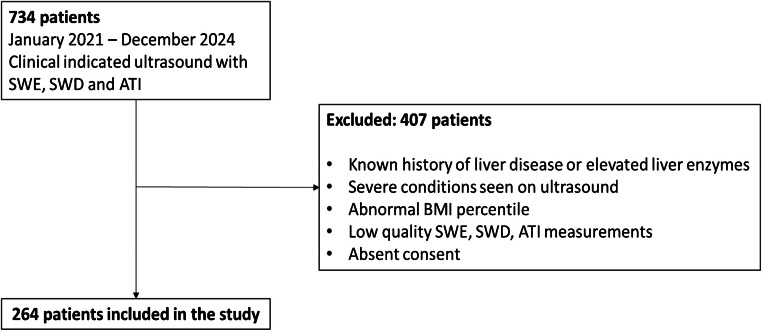
Flowchart illustrating the patient inclusion process*. ATI* attenuation imaging coefficient*, BMI* body mass index*, SWD* shear wave dispersion*, SWE* shear wave elastography

A total of 734 children who underwent clinically indicated ultrasound examinations with measurements of shear wave elastography, shear wave dispersion, and attenuation imaging between January 2021 and December 2024 were initially analysed. From this cohort, 264 patients were enrolled in the study based on the following inclusion criteria: under 18 years of age, no known history of liver disease or elevated liver enzymes, no severe conditions identified in ultrasound, normal organ sizes, normal BMI percentiles (2.5th to 97.5th), good-quality measurements of shear wave elastography, shear wave dispersion, and attenuation imaging, and consent for participation and the use of their data for research purposes (Fig. 1).

Age, height, weight, and fasting duration prior to the ultrasound examination were extracted from the patient archiving system, with fasting recorded in hours or marked as unknown if not documented in the report.

### Ultrasound examination

Abdominal ultrasound was performed using an Aplio i800 (Canon Medical Systems, Otawara, Japan) with an i8CX1 transducer (PVI- 475BX, iDMS single crystal active-matrix curved array, 1.8–6.2 MHz). The ultrasound procedures were carried out by a team of six experienced paediatric radiologists (between 11- and 31-year experience in paediatric radiology) skilled in using the Canon system and quantitative liver ultrasound, with all images acquired in accordance with a standardized protocol. Children were examined in a supine position with their arms raised above the head, and measurements were taken during a breath-hold at neutral breathing whenever possible. Those unable to hold their breath due to age were assessed during free breathing.

### Attenuation imaging coefficient

**Fig. 2:**
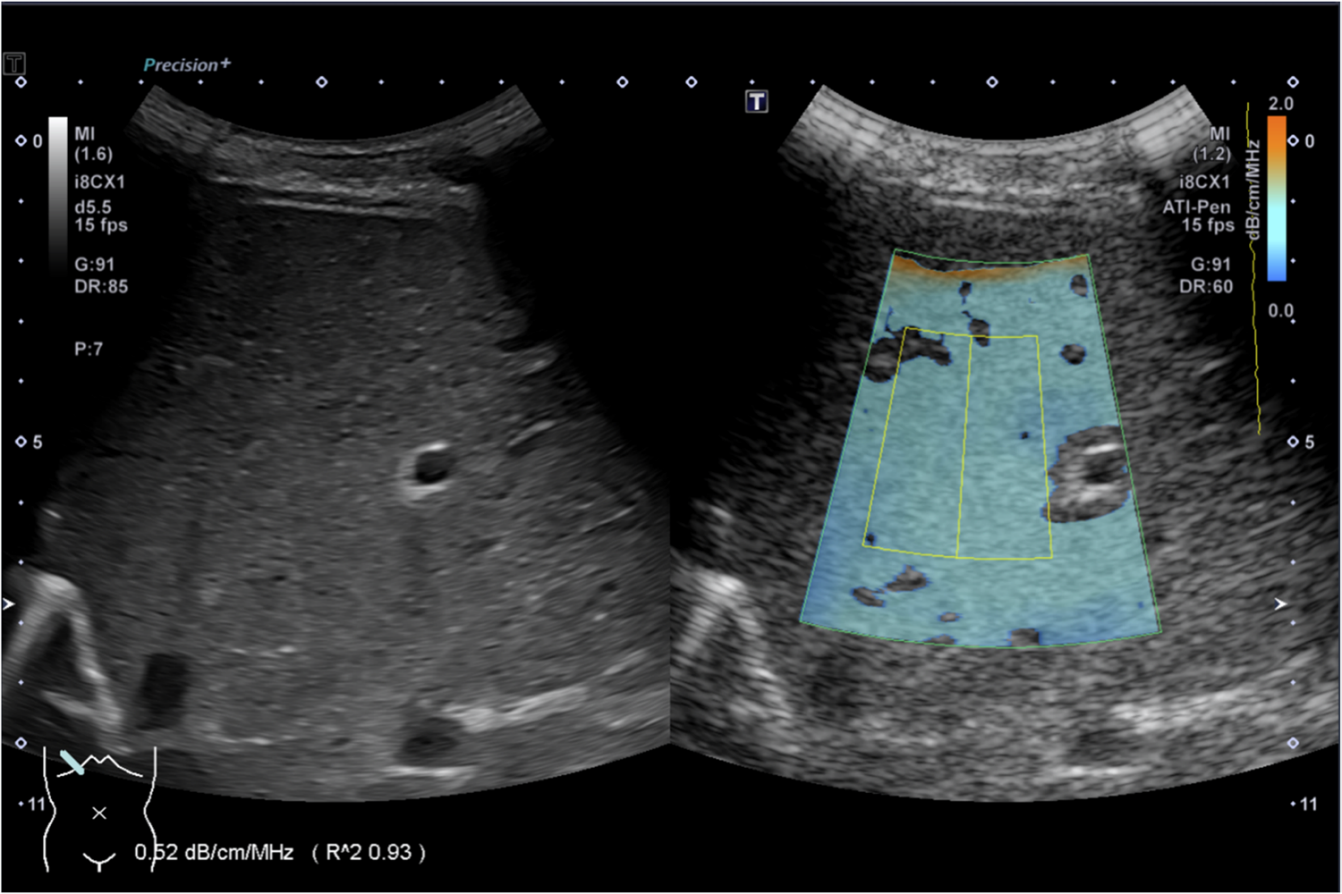
Representative attenuation imaging coefficient measurement (liver ultrasound in the transverse intercostal plane) in a 14-year-old healthy boy with an attenuation imaging coefficient of 0.52 dB/cm/MHz and an *R*^2^ of 0.93

Attenuation imaging was measured using an intercostal approach, with five measurements taken while avoiding areas near the liver capsule (Fig. 2). Only measurements with an excellent goodness-of-fit (*R*^2^ > 0.90) were accepted. However, in neonates, due to their smaller liver size, an *R*^2^ > 0.80 was considered acceptable. The median and interquartile range (IQR) of these five measurements were extracted from the score sheet.

### Shear wave elastography and dispersion

**Fig. 3:**
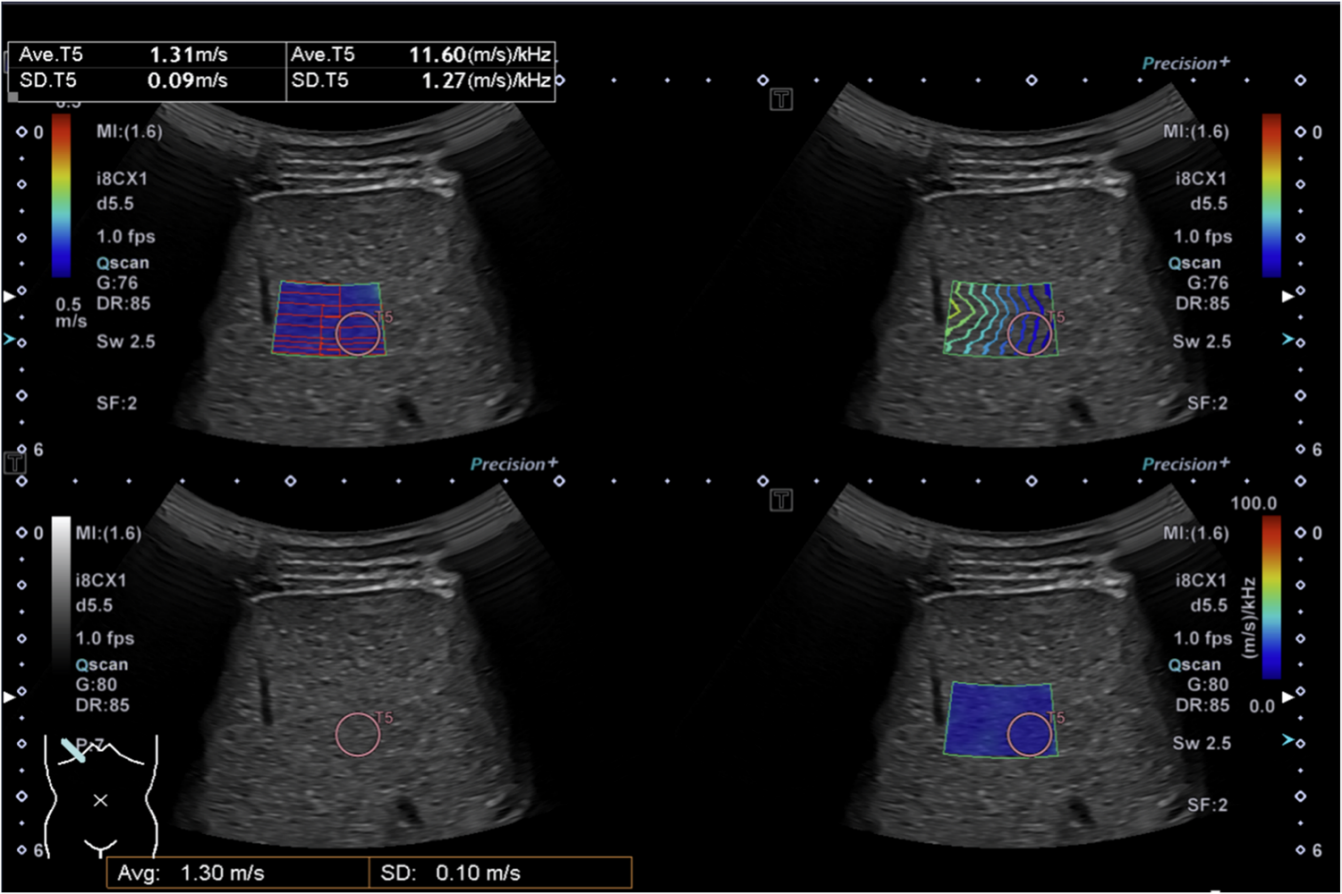
Representative quad view measurement number five out of 12 (liver ultrasound in the transverse intercostal plane) in a 14-year-old healthy boy with a shear wave elasticity of 1.31 m/s and a shear wave dispersion of 11.60 (m/s)/kHz

Shear wave elastography and shear wave dispersion were measured in accordance with the World Federation for Ultrasound in Medicine and Biology guidelines [20]. A quad-view display with multi-shot mode was used to ensure a well-defined propagation map within a 1-cm region of interest at a depth of 3 cm to 5 cm below the skin (Fig. 3). Following this protocol, ten measurements were obtained from the right liver lobe (segments V–VIII). The median and interquartile range (IQR) of these ten measurements were extracted from the score sheet. Only measurements meeting the criteria of IQR/median ≤ 30% for kPa and ≤ 15% for m/s were included to ensure an accurate dataset. Additionally, the depth of each measurement was recorded for further evaluation. In cases where shear wave speed or elasticity was not provided in the literature, values were estimated using the Young’s modulus equation (*E* = 3ρc^2^), assuming the liver behaves as an incompressible, isotropic, elastic medium with a tissue density (*ρ*) of approximately 1,000 kg/m^3^.

### Statistical analysis

Continuous variables were given as means and standard deviations or medians and interquartile ranges. Categorical variables were presented as counts and percentages. Univariate analyses were performed to explore associations between patient-specific variables and each quantitative liver measure: shear wave elastography, shear wave dispersion, and attenuation imaging. Sex differences were assessed using a two-sided *t*-test, while associations for continuous predictor variables, including age, height, weight, body mass index (BMI), height *z* scores, weight *z* scores, BMI *z* scores, and measurement depth were analysed using Pearson correlation coefficients. Correlation strength was categorized as follows: very weak (*r *= 0–0.19), weak (*r* = 0.20–0.39), moderate (*r* = 0.40–0.59), strong (*r* = 0.60–0.79), and very strong (*r* = 0.80–1.00) [21]. Differences in quantitative liver measures between participants with fasting durations of at least 4 h versus less than 4 h were assessed using the Wilcoxon rank-sum test. *P*-values were adjusted with the Benjamini–Hochberg procedure for multiple comparisons. A two-tailed *P-*value less than 0.05 was considered to indicate statistical significance.

In the study by Cetiner et al. [6], age was identified as the main predictor of attenuation imaging in healthy children. Following their approach, we constructed a reference model for attenuation imaging using generalized additive models for location, scale, and shape (GAMLSS) [22]. The Box-Cox Cole and Green (BCCG) distribution [23] was applied, in which its distribution parameters median (*μ*), coefficient of variation (*σ*), and skewness (*ν*) were modelled as functions of age. Model selection within GAMLSS was performed via an exhaustive grid search, testing different functional forms of age for each distribution parameter. Specifically, for each parameter, age was either excluded, included as a linear term, or modelled as a nonlinear smooth term using cubic splines with degrees of freedom ranging from 1 to 5. All combinations of predictor forms were evaluated independently for each of the distribution parameters (*μ*, *σ*, and *ν*). The model configuration with the lowest Bayesian Information Criterion (BIC) was selected as the final model [22]. We restricted this modelling approach to attenuation imaging only, as Cetiner et al. [6] demonstrated a clear age dependency for attenuation imaging, making age-specific centile curves clinically practical and relevant.

All analyses were performed in SPSS Statistics Version 26 (IBM Corp., Armonk, NY) and R (versions 4.4.3). GAMLSS models were fitted using the gamlss package (version 5.4–22).

## Results

### Patients

In total, 264 patients (135 female) median age 11.53 years (range 0.13 years to 17.91 years) were included in the study (Table 1). The median height of the study cohort was 147.65 cm (range 47–183.80 cm), and the median weight was 40.60 kg (range 2.27–84.00 kg). No significant differences were observed between the 5th–85th and 2.5th–97.5th BMI percentile groups for ATI, SWE, or SWD (*t*-test, FDR-adjusted *P* > 0.05). The subgroup analysis for BMI percentiles is presented in Supplementary material 1. Fasting duration prior to the ultrasound examination was undocumented in 125 cases, while 41 children fasted for more than 4 h, 96 for less than 4 h, and 97 for more than 2 h. The overall median fasting time was 2 h. No significant differences were observed between the groups fasting for more than 4 h and those fasting for less than 4 h across all measurements (Wilcoxon test, FDR adjusted *P* > 0.05).

**Table 1:** Patient characteristics divided by age groups

		BMI		BMI percentile		SWE (m/s)		SWE (kPa)		SWD ((m/s)/kHz)		ATI (dB/cm/MHz)	
		Mean ± SD	Median [IQR]	Mean ± SD	Median [IQR]	Mean ± SD	Median [IQR]	Mean ± SD	Median [IQR]	Mean ± SD	Median [IQR]	Mean ± SD	Median [IQR]
All patients	0 to 18-year-old (*n* = 264)	18.41 ± 2.87	17.82 [16.17- 20.70]	52.99 ± 26.86	56.10 [31.70- 76.15]	1.24 ± 0.13	1.24 [1.14- 1.33]	4.73 ± 0.91	4.70 [4.20- 5.30]	11.59 ± 1.29	11.70 [10.84- 12.13]	0.53 ± 0.07	0.54 [0.50- 0.58]
Age groups	0 to 2-year-old (*n* = 18)	15.78 ± 2.09	16.20 [14.99- 16.99]	46.79 ± 32.04	49.85 [14.00- 72.90]	1.26 ± 0.13	1.23 [1.18- 1.33]	4.83 ± 1.00	4.55 [4.20- 5.30]	12.40 ± 1.21	11.99 [11.70- 12.90]	0.58 ± 0.07	0.57 [0.54- 0.61]
	2 to 5-year-old (*n* = 23)	15.98 ± 1.15	15.97 [15.37- 16.61]	57.72 ± 24.05	58.00 [48.10- 71.80]	1.26 ± 0.09	1.26 [1.18- 1.33]	4.77 ± 0.70	4.80 [4.20- 5.30]	11.74 ± 1.08	11.70 [11.30- 12.30]	0.55 ± 0.05	0.56 [0.53- 0.59]
	5 to 10-year-old (*n* = 65)	16.33 ± 1.72	16.38 [15.14- 17.35]	54.22 ± 26.47	60.20 [36.80- 73.60]	1.24 ± 0.12	1.24 [1.18- 1.30]	4.64 ± 0.86	4.60 [4.20- 5.10]	11.82 ± 1.02	11.90 [11.10- 12.40]	0.53 ± 0.08	0.54 [0.50- 0.57]
	10 to 15-year-old (*n* = 95)	19.18 ± 2.41	19.56 [17.16- 20.83]	54.00 ± 27.40	55.20 [32.30- 79.40]	1.25 ± 0.13	1.25 [1.15- 1.34]	4.75 ± 0.99	4.70 [4.00- 5.40]	11.38 ± 1.23	11.53 [10.59- 12.10]	0.53 ± 0.06	0.53 [0.50- 0.57]
	15-year-old and above (*n* = 63)	21.04 ± 2.18	21.13 [19.52- 22.60]	50.25 ± 26.10	55.50 [29.20- 74.90]	1.25 ± 0.12	1.25 [1.18- 1.33]	4.75 ± 0.87	4.70 [4.20- 5.30]	11.40 ± 1.59	11.40 [10.40- 12.10]	0.52 ± 0.06	0.53 [0.49- 0.57]
Sex	Female (*n* = 135)	18.73 ± 2.89	18.89 [16.42- 20.87]	53.50 ± 25.63	56.10 [35.70- 76.30]	1.26 ± 0.11	1.26 [1.20- 1.33]	4.68 ± 0.99	4.60 [3.90- 5.30]	11.32 ± 1.35	11.50 [10.50- 12.03]	0.54 ± 0.06	0.54 [0.50- 0.57]
	Male (*n* = 129)	18.08 ± 2.82	17.35 [16.05- 20.26]	52.46 ± 28.18	56.10 [29.40- 76.10]	1.25 ± 0.12	1.25 [1.18- 1.33]	4.79 ± 0.81	4.80 [4.30- 5.30]	11.88 ± 1.17	11.89 [11.10- 12.40]	0.53 ± 0.08	0.54 [0.49- 0.59]

Data is presented as mean ± standard deviation or as median [interquartile range]

*ATI* attenuation imaging coefficient, *BMI* body mass index, *IQR* interquartile range, *n* number of patients, *SD* standard deviation, *SWD* shear wave dispersion, *SWE* shear wave elastography

### Attenuation imaging coefficient

A total of 1,320 attenuation imaging measurements were analysed across 264 patients. The overall median attenuation imaging was 0.54 dB/cm/MHz (IQR 0.50–0.58). Among age groups, children aged 0 years to 2 years exhibited the highest median attenuation imaging, measuring 0.57 dB/cm/MHz (IQR 0.54–0.61) (Table 1).

A significant negative correlation was observed between attenuation imaging and age (*r* = –0.23, 95% CI –0.34 to –0.12; FDR adjusted *P* < 0.001). No other patient characteristics, including sex, showed a statistically significant association with attenuation imaging.

**Fig. 4:**
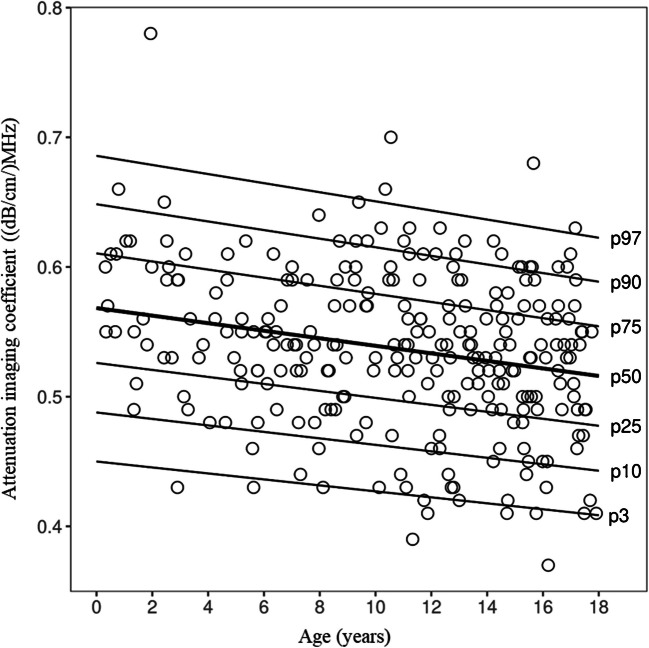
Estimated centile curves for attenuation imaging in healthy children derived from the final generalized additive models for location, scale, and shape with a Box-Cox Cole and Green distribution. The 3rd, 10th, 25th, 50th, 75th, 90th, and 97th percentiles are shown as functions of age. The *dots* show individual attenuation imaging measurements

The final GAMLSS model included age as a linear predictor of the median (*μ*), with constant *σ* and *ν*. Each additional year of age was associated with a −0.003 dB/cm/MHz decrease in the median attenuation imaging (95% CI −0.004 to −0.001). Age-dependent centile curves are shown in Fig. 4, and corresponding centile values for selected ages are provided in Supplementary material 2 for clinical interpretation.

### Shear wave elastography and dispersion

A total of 2,640 shear wave elastography and shear wave dispersion measurements were analyzed. The median shear wave elastography was 1.24 m/s (IQR 1.14–1.33), and the median shear wave dispersion was 11.70 (m/s)/kHz (IQR 10.84–12.13).

**Fig. 5:**
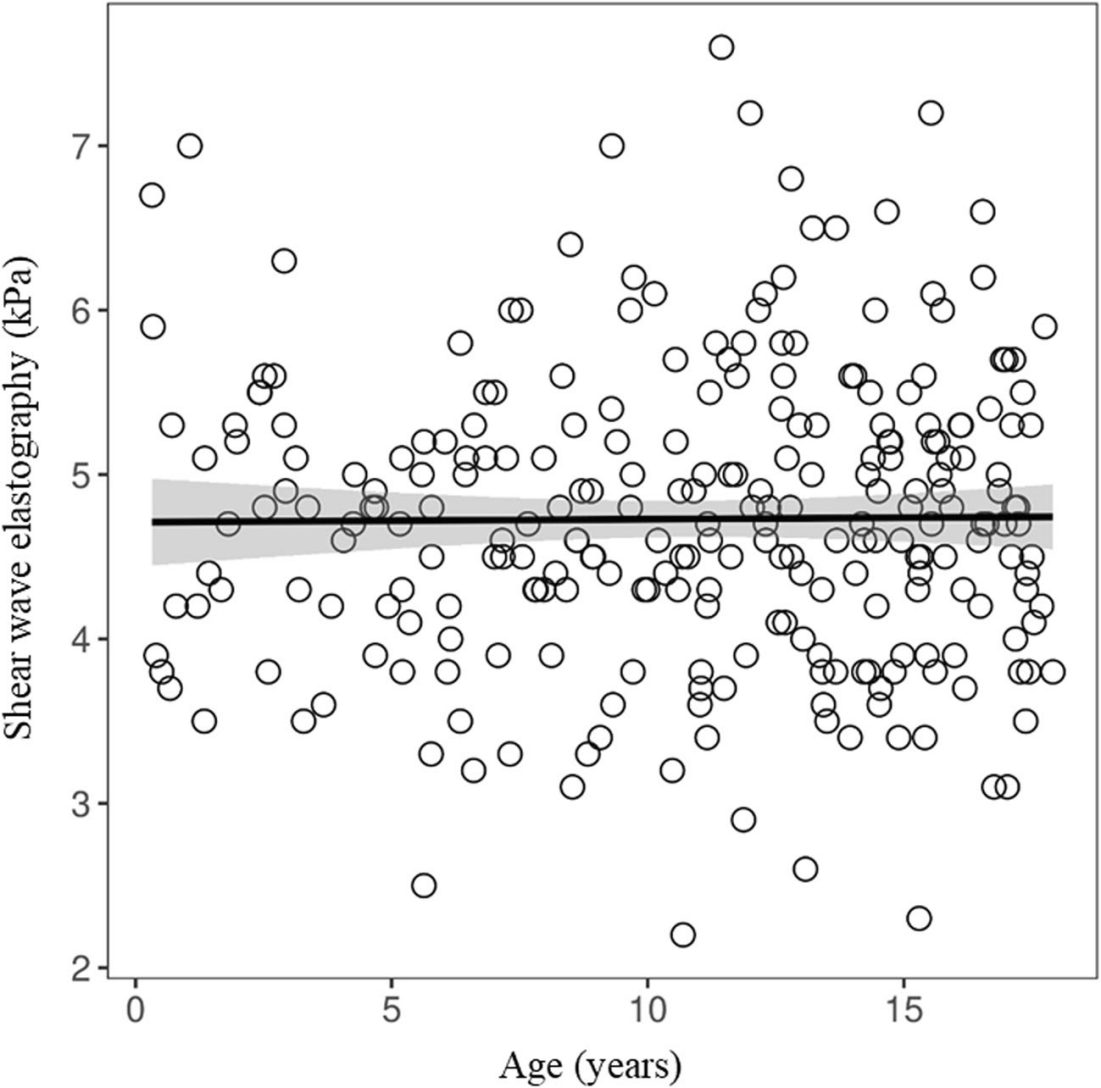
Scatter plot illustrating the relationship between shear wave elastography and age, demonstrating no correlation. *SWE*, shear wave elastography

Shear wave elastography showed a very weak positive correlation with measurement depth (*r* = 0.17, 95% CI 0.05 to 0.29; FDR adjusted *P* = 0.0261), while no other predictors, especially age, were associated with shear wave elastography (Fig. 5).

**Fig. 6:**
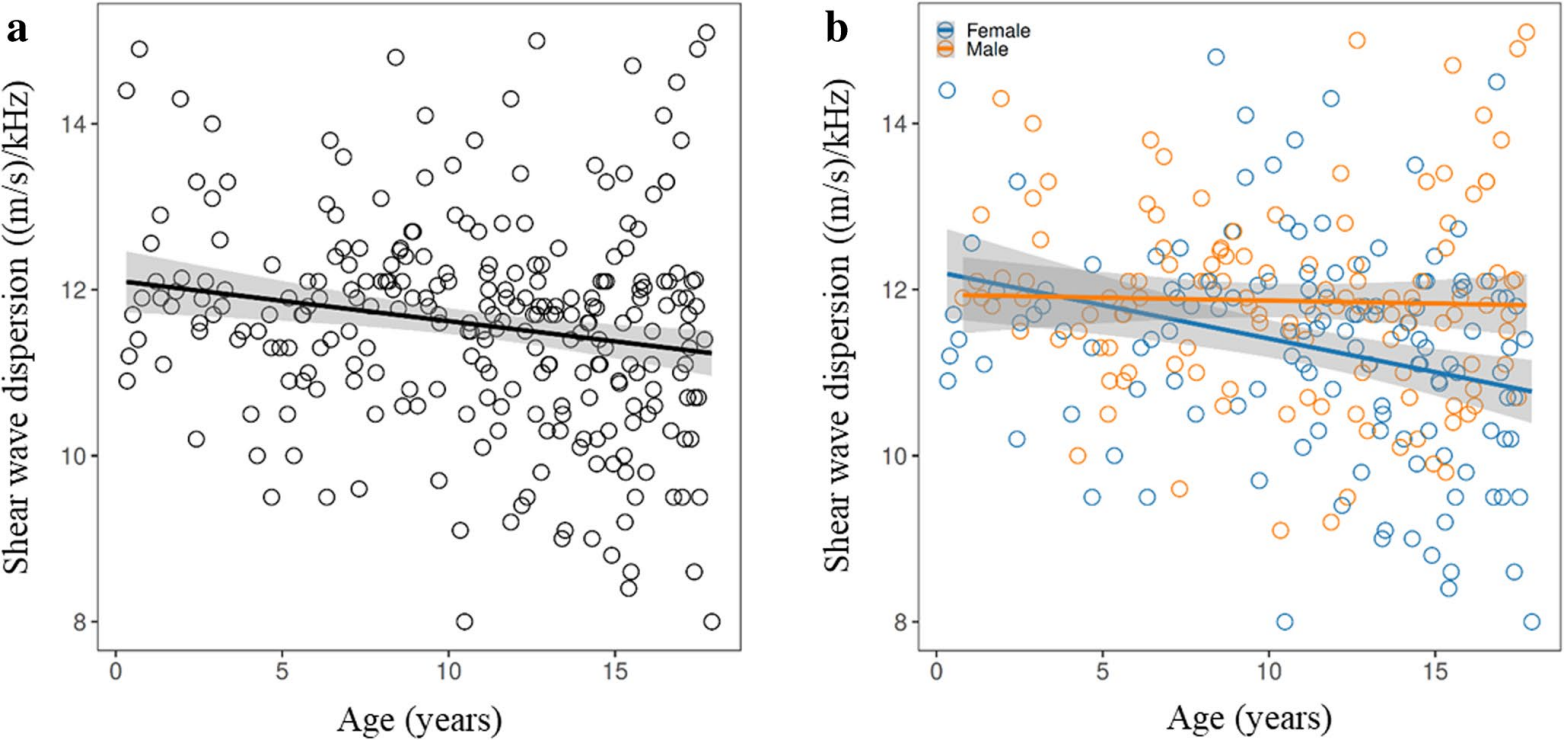
**a**, **b** Scatter plots illustrating the relationship between shear wave dispersion, age and sex. Shear wave dispersion showed negative correlations with age **a** and was significantly lower in females compared to males **b**

Shear wave dispersion was significantly lower in females compared to males (difference: –0.56 (m/s)/kHz, 95% CI:–0.87 to –0.26; FDR adjusted *P* = 0.001). Shear wave dispersion was also negatively correlated with age (*r* = –0.20, 95% CI –0.31 to –0.08; FDR adjusted *P* = 0.0048) and with BMI *z*-score (*r *= –0.32, 95% CI –0.42 to –0.21; FDR adjusted *P* < 0.001) (Fig. 6).

## Discussion

This study aimed to establish normal reference values for the non-invasive techniques shear wave elastography, shear wave dispersion, and attenuation imaging in healthy children, taking into account potential influencing factors. Only a limited number of studies have investigated this emerging ultrasound technique in healthy children (Table 2). To date, only two studies—Sarovic et al. (65 children) and Cetiner et al. (112 children)—have comprehensively evaluated all three ultrasound modalities (shear wave elastography, shear wave dispersion, and attenuation imaging) in this population [5, 6].

**Table 2:** Overview of previous liver ultrasound elastography and attenuation studies in healthy children

First author year [reference]	Technique	Ultrasound system	Children in *n*	SWE (kPa)	SWE (m/s)	SWD ((m/s)/kHz)	ATI (dB/cm/MHz)
Sarovic 2024 [5]	ATI, SWE, SWD	Canon® Aplio i800	65	4.9 ± 1.1	NA	11.9 ± 1.5	0.56 ± 0.09
Sook 2023 [19]	ATI	Canon® Aplio i900	40	NA	NA	NA	0.50 ± 0.10
Cetiner 2023 [6]	ATI, SWE, SWD	Canon® Aplio i800	112	4.37 ± 0.60	1.22 ± 0.08	12.96 ± 1.52	0.59 ± 0.07
Cailloce 2021 [17]	ATI, SWE	Canon® Aplio i800	86	4.6 ± 0.60	NA	NA	0.65 ± 0.07
Trout 2020 [16]	SWE, SWD	Canon® Aplio i800	128	NA	1.29 ± 0.13	11.43 ± 1.75	NA
Mjelle 2019 [30]	SWE	General Electric® Logiq E9 US	243	3.3 ± 0.60	NA	NA	NA
Galina 2019 [18]	SWE	General Electric® Logiq E9 US	202	4.29 ± 0.59	NA	NA	NA

Data is presented as mean ± standard deviation

*ATI* attenuation imaging coefficient, *NA* not available, *SWD* shear wave dispersion, *SWE* shear wave elastography

In this study, we intentionally included children with varying fasting durations to assess whether this factor influenced the results. Extended fasting, particularly in infants, may reduce cooperation during the examination and hinder its success. However, multivariable analysis did not reveal any significant association between fasting duration and shear wave elastography, shear wave dispersion, or attenuation imaging measurements, suggesting that the length of fasting has minimal impact on these quantitative ultrasound parameters. This finding is in line with previous studies by Cetiner et al. and Paverd et al. both of whom also concluded that the prandial state does not significantly affect these measurements and can therefore be considered negligible [6, 24].

The mean attenuation imaging value of 0.53 dB/cm/MHz observed in this study aligns well with published normative data, with most studies reporting normal mean attenuation imaging values in children without steatosis ranging from approximately 0.50 dB/cm/MHz to 0.56 dB/cm/MHz [5, 6, 19]. An exception to the general trend is the study by Cailloce et al. [17], which reported a median attenuation imaging value of 0.65 dB/cm/MHz in 86 children without clinically suspected liver disease. This value exceeds typical adult reference values (0.52 dB/cm/MHz) [16, 25] and falls within the range associated with mild hepatic steatosis in adults (0.63–0.70 dB/cm/MHz) [26]. Their methodology included only two attenuation imaging measurements per subject, whereas five measurements were taken in the present study, potentially reducing measurement variability. Cailloce et al. [17] speculated that a thickened hepatocyte layer in children under 5 years might contribute to elevated attenuation imaging values, though no age-stratified analysis was conducted to support this hypothesis. This study showed similar findings to Cetiner et al. that attenuation imaging has a significant negative correlation to age, indicating that there is a decrease in attenuation imaging during childhood (Fig. 4) [6]. One proposed explanation for age-related variations in attenuation imaging values involves developmental differences in liver structure and fat content. Younger children, particularly infants and preschoolers, may naturally have higher hepatic fat levels, which would likely influence attenuation imaging measurements. Additionally, early-life factors such as prenatal growth patterns and infant nutrition may contribute to variations in liver fat content observed in school-aged children [27, 28].

The GAMLSS model showed a systematic decrease in attenuation imaging with age, estimating a reduction of 0.003 (dB/cm)/MHz per year in the median attenuation imaging. Over an 18-year period, this corresponds to a cumulative decrease of approximately 0.054 (dB/cm)/MHz, which is nearly equivalent to the overall inter-individual variability observed in our cohort (SD≈0.06). This highlights the practical relevance of age adjustment and supports the use of age-specific centile curves for clinical interpretation. Compared to the centile values reported by Cetiner et al. [6], our predicted attenuation imaging centiles were systematically lower across early childhood. For example, at age 2 years, the 3rd, 50th, and 97th percentiles were estimated at 0.45 (dB/cm)/MHz, 0.56 (dB/cm)/MHz, and 0.68 (dB/cm)/MHz in our cohort, compared to 0.54 (dB/cm)/MHz, 0.63 (dB/cm)/MHz, and 0.72 (dB/cm)/MHz, respectively, in the study by Cetiner et al. [6]. These differences were consistent across age groups and percentiles and are likely explained by differences in measurement protocols or population characteristics. Nevertheless, both studies demonstrate a clear age dependency in attenuation imaging, reinforcing the need for age-adjusted centile curves. To maintain clinical practicality, we limited the model to age as the sole predictor of attenuation imaging, consistent with the approach used by Cetiner et al. Other factors related to body composition or physiology may also play a role but were not included to avoid increasing model complexity and limiting its applicability in routine practice.

This study reported a mean shear wave elastography of 1.24 ± 0.13 m/s, which is comparable to values reported by Cetiner (1.22 ± 0.08 m/s) and Trout (1.29 ± 0.13 m/s) [6, 16]. Other studies that reported shear wave elastography values in kPa rather than m/s showed comparable results, with converted values ranging from 1.05 ± 0.45 m/s (Mjelle et al.) to 1.28 ± 0.61 m/s (Sarovic et al.) (Table 2). Interestingly, and in contrast to the findings of Cetiner and Galina, but in agreement with Trout, no significant correlation was observed between shear wave elastography and either age (Fig. 5) or sex [6, 16, 18]. Only a very weak correlation with measurement depth was detected. The observed depth dependency highlights the importance of adhering to the World Federation for Ultrasound in Medicine and Biology guidelines for proper performance of quantitative ultrasound measurements [20].

The mean shear wave dispersion value in this study was 11.59 ± 1.29 (m/s)/kHz, which is consistent with the limited existing literature on normal shear wave dispersion values in healthy paediatric populations. Sarovic et al. reported a mean shear wave dispersion of 11.9 ± 1.5 (m/s)/kHz, Cetiner et al. reported 12.96 ± 1.52 (m/s)/kHz, and Trout et al. found 11.43 ± 1.75 (m/s)/kHz (Table 2) [5, 6, 16]. Normative values observed in children tend to be higher than those reported in adults, which may be due to age-related variations in tissue viscoelasticity [16, 29]. In contrast to previous literature, this study identified a moderate negative correlation between shear wave dispersion values and age. Furthermore, shear wave dispersion was found to be significantly lower in females compared to males (Fig. 6). Unlike shear wave elastography, shear wave dispersion values also showed a weak negative correlation with subjects’ BMI *z*-scores, a finding that is consistent with the observations reported by Cetiner et al. [6].

Several limitations of this study merit consideration. First, the retrospective design may introduce selection and information bias. Second, although the population was thoroughly screened for liver pathology, some participants may have had minor illnesses that could have influenced liver viscoelasticity. Additionally, the number of children who were fasting (nothing per os) for more than 4 h was low; however, this limitation is likely negligible given the inconsistent evidence in the literature regarding the effect of fasting on attenuation imaging, shear wave elastography, and shear wave dispersion values. Another limitation is that the respiratory state during image acquisition was not recorded. Lastly, the study population was predominantly white, which may limit the generalizability of the findings to more ethnically diverse populations.

## Conclusion

This study provides age-specific reference values for shear wave elastography, shear wave dispersion, and attenuation imaging in healthy children. Attenuation imaging showed a clear age dependency, and age-adjusted centile curves were developed to aid clinical interpretation. Shear wave elastography remained consistent across age and sex, indicating its reliability as an age-independent parameter. In contrast, shear wave dispersion was lower in females and negatively associated with age and BMI *z*-score. These findings highlight the importance of considering age when interpreting attenuation imaging and sex when assessing shear wave dispersion, while supporting the robustness of shear wave elastography across paediatric subgroups.

## Supplementary information

Below is the link to the electronic supplementary material.

Supplementary file 1 (DOCX 41.8 KB)

## Data Availability

Anonymized data supporting the findings of this study are available from the corresponding author upon reasonable request. In addition, Dataset 1 (SPSS file) has been uploaded as supplementary material and is available to reviewers during the peer review process.
