# Supplementary material for: Reference values for shear wave elastography, shear wave dispersion and attenuation imaging in healthy paediatric livers
Source: Pediatr Radiol. 2025 Nov 1;56(1):179–89. doi: 10.1007/s00247-025-06434-9 (PMC12831688; doi:10.1007/s00247-025-06434-9)
Supplement: Supplementary file 1 — (DOCX 41.8 KB) [file 247_2025_6434_MOESM1_ESM.docx]

# **Reference values for shear wave elastography, shear wave dispersion and attenuation imaging in healthy paediatric livers**

# *Original research*

# Supplementary material

**Supplementary material 1:** Distribution and quantitative measurements of patients by body mass index percentiles.

|  | Number of patients | SWE (m/s) | SWE (kPa) | SWD ((m/s)/kHz) | ATI (dB/cm/MHz) |  |
| --- | --- | --- | --- | --- | --- | --- |
|  |  |  |  |  |  |  |
| BMI percentile<5^th^ | 10 | 1.28±0.08 | 4.96±0.68 | 12.67±1.10 | 0.51±0.05 |  |
| BMI 5^th^ to 85^th^ | 219 | 1.25±0.12 | 4.75±0.92 | 11.62±1.27 | 0.54±0.06 |  |
| BMI percentile>85^th^ | 35 | 1.22±0.12 | 4.53±0.89 | 11.14±1.32 | 0.54±0.07 |  |
| BMI 2.5^th^ to 97.5^th^ | 264 | 1.24±0.13 | 4.73±0.91 | 11.59±1.29 | 0.53±0.07 |  |

*ATI* attenuation imaging coefficient, *BMI* body mass index, *SWD* shear wave dispersion, *SWE* shear wave elastography. Data is presented as mean ± standard deviation

**Supplementary material 2:** Age-specific predicted centile values for attenuation imaging derived from the final generalized additive models for location, scale, and shape. The table presents the estimated 3rd, 10th, 25th, 50th, 75th, 90th, and 97th percentiles of attenuation imaging (dB/cm/MHz) as a function of age in healthy children.

| Age (years) | 3rd percentile | 10th percentile | 25th percentile | 50th percentile | 75th percentile | 90th percentile | 97th percentile |
| --- | --- | --- | --- | --- | --- | --- | --- |
| 0 | 0.45 | 0.49 | 0.53 | 0.57 | 0.61 | 0.65 | 0.69 |
| 0.2 | 0.45 | 0.49 | 0.53 | 0.57 | 0.61 | 0.65 | 0.68 |
| 0.4 | 0.45 | 0.49 | 0.52 | 0.57 | 0.61 | 0.65 | 0.68 |
| 0.6 | 0.45 | 0.49 | 0.52 | 0.57 | 0.61 | 0.65 | 0.68 |
| 0.8 | 0.45 | 0.49 | 0.52 | 0.57 | 0.61 | 0.65 | 0.68 |
| 1 | 0.45 | 0.49 | 0.52 | 0.57 | 0.61 | 0.65 | 0.68 |
| 1.2 | 0.45 | 0.48 | 0.52 | 0.56 | 0.61 | 0.64 | 0.68 |
| 1.4 | 0.45 | 0.48 | 0.52 | 0.56 | 0.61 | 0.64 | 0.68 |
| 1.6 | 0.45 | 0.48 | 0.52 | 0.56 | 0.61 | 0.64 | 0.68 |
| 1.8 | 0.45 | 0.48 | 0.52 | 0.56 | 0.60 | 0.64 | 0.68 |
| 2 | 0.45 | 0.48 | 0.52 | 0.56 | 0.60 | 0.64 | 0.68 |
| 2.2 | 0.44 | 0.48 | 0.52 | 0.56 | 0.60 | 0.64 | 0.68 |
| 2.4 | 0.44 | 0.48 | 0.52 | 0.56 | 0.60 | 0.64 | 0.68 |
| 2.6 | 0.44 | 0.48 | 0.52 | 0.56 | 0.60 | 0.64 | 0.68 |
| 2.8 | 0.44 | 0.48 | 0.52 | 0.56 | 0.60 | 0.64 | 0.68 |
| 3 | 0.44 | 0.48 | 0.52 | 0.56 | 0.60 | 0.64 | 0.68 |
| 3.2 | 0.44 | 0.48 | 0.52 | 0.56 | 0.60 | 0.64 | 0.67 |
| 3.4 | 0.44 | 0.48 | 0.52 | 0.56 | 0.60 | 0.64 | 0.67 |
| 3.6 | 0.44 | 0.48 | 0.52 | 0.56 | 0.60 | 0.64 | 0.67 |
| 3.8 | 0.44 | 0.48 | 0.52 | 0.56 | 0.60 | 0.64 | 0.67 |
| 4 | 0.44 | 0.48 | 0.52 | 0.56 | 0.60 | 0.64 | 0.67 |
| 4.2 | 0.44 | 0.48 | 0.51 | 0.56 | 0.60 | 0.63 | 0.67 |
| 4.4 | 0.44 | 0.48 | 0.51 | 0.56 | 0.60 | 0.63 | 0.67 |
| 4.6 | 0.44 | 0.48 | 0.51 | 0.55 | 0.60 | 0.63 | 0.67 |
| 4.8 | 0.44 | 0.48 | 0.51 | 0.55 | 0.60 | 0.63 | 0.67 |
| 5 | 0.44 | 0.48 | 0.51 | 0.55 | 0.59 | 0.63 | 0.67 |
| 5.2 | 0.44 | 0.47 | 0.51 | 0.55 | 0.59 | 0.63 | 0.67 |
| 5.4 | 0.44 | 0.47 | 0.51 | 0.55 | 0.59 | 0.63 | 0.67 |
| 5.6 | 0.44 | 0.47 | 0.51 | 0.55 | 0.59 | 0.63 | 0.67 |
| 5.8 | 0.44 | 0.47 | 0.51 | 0.55 | 0.59 | 0.63 | 0.67 |
| 6 | 0.44 | 0.47 | 0.51 | 0.55 | 0.59 | 0.63 | 0.66 |
| 6.2 | 0.44 | 0.47 | 0.51 | 0.55 | 0.59 | 0.63 | 0.66 |
| 6.4 | 0.44 | 0.47 | 0.51 | 0.55 | 0.59 | 0.63 | 0.66 |
| 6.6 | 0.43 | 0.47 | 0.51 | 0.55 | 0.59 | 0.63 | 0.66 |
| 6.8 | 0.43 | 0.47 | 0.51 | 0.55 | 0.59 | 0.63 | 0.66 |
| 7 | 0.43 | 0.47 | 0.51 | 0.55 | 0.59 | 0.63 | 0.66 |
| 7.2 | 0.43 | 0.47 | 0.51 | 0.55 | 0.59 | 0.62 | 0.66 |
| 7.4 | 0.43 | 0.47 | 0.51 | 0.55 | 0.59 | 0.62 | 0.66 |
| 7.6 | 0.43 | 0.47 | 0.51 | 0.55 | 0.59 | 0.62 | 0.66 |
| 7.8 | 0.43 | 0.47 | 0.50 | 0.55 | 0.59 | 0.62 | 0.66 |
| 8 | 0.43 | 0.47 | 0.50 | 0.55 | 0.59 | 0.62 | 0.66 |
| 8.2 | 0.43 | 0.47 | 0.50 | 0.54 | 0.58 | 0.62 | 0.66 |
| 8.4 | 0.43 | 0.47 | 0.50 | 0.54 | 0.58 | 0.62 | 0.66 |
| 8.6 | 0.43 | 0.47 | 0.50 | 0.54 | 0.58 | 0.62 | 0.66 |
| 8.8 | 0.43 | 0.47 | 0.50 | 0.54 | 0.58 | 0.62 | 0.65 |
| 9 | 0.43 | 0.47 | 0.50 | 0.54 | 0.58 | 0.62 | 0.65 |
| 9.2 | 0.43 | 0.46 | 0.50 | 0.54 | 0.58 | 0.62 | 0.65 |
| 9.4 | 0.43 | 0.46 | 0.50 | 0.54 | 0.58 | 0.62 | 0.65 |
| 9.6 | 0.43 | 0.46 | 0.50 | 0.54 | 0.58 | 0.62 | 0.65 |
| 9.8 | 0.43 | 0.46 | 0.50 | 0.54 | 0.58 | 0.62 | 0.65 |
| 10 | 0.43 | 0.46 | 0.50 | 0.54 | 0.58 | 0.62 | 0.65 |
| 10.2 | 0.43 | 0.46 | 0.50 | 0.54 | 0.58 | 0.61 | 0.65 |
| 10.4 | 0.43 | 0.46 | 0.50 | 0.54 | 0.58 | 0.61 | 0.65 |
| 10.6 | 0.43 | 0.46 | 0.50 | 0.54 | 0.58 | 0.61 | 0.65 |
| 10.8 | 0.43 | 0.46 | 0.50 | 0.54 | 0.58 | 0.61 | 0.65 |
| 11 | 0.42 | 0.46 | 0.50 | 0.54 | 0.58 | 0.61 | 0.65 |
| 11.2 | 0.42 | 0.46 | 0.50 | 0.54 | 0.58 | 0.61 | 0.65 |
| 11.4 | 0.42 | 0.46 | 0.50 | 0.54 | 0.57 | 0.61 | 0.65 |
| 11.6 | 0.42 | 0.46 | 0.49 | 0.53 | 0.57 | 0.61 | 0.64 |
| 11.8 | 0.42 | 0.46 | 0.49 | 0.53 | 0.57 | 0.61 | 0.64 |
| 12 | 0.42 | 0.46 | 0.49 | 0.53 | 0.57 | 0.61 | 0.64 |
| 12.2 | 0.42 | 0.46 | 0.49 | 0.53 | 0.57 | 0.61 | 0.64 |
| 12.4 | 0.42 | 0.46 | 0.49 | 0.53 | 0.57 | 0.61 | 0.64 |
| 12.6 | 0.42 | 0.46 | 0.49 | 0.53 | 0.57 | 0.61 | 0.64 |
| 12.8 | 0.42 | 0.46 | 0.49 | 0.53 | 0.57 | 0.61 | 0.64 |
| 13 | 0.42 | 0.46 | 0.49 | 0.53 | 0.57 | 0.61 | 0.64 |
| 13.2 | 0.42 | 0.45 | 0.49 | 0.53 | 0.57 | 0.60 | 0.64 |
| 13.4 | 0.42 | 0.45 | 0.49 | 0.53 | 0.57 | 0.60 | 0.64 |
| 13.6 | 0.42 | 0.45 | 0.49 | 0.53 | 0.57 | 0.60 | 0.64 |
| 13.8 | 0.42 | 0.45 | 0.49 | 0.53 | 0.57 | 0.60 | 0.64 |
| 14 | 0.42 | 0.45 | 0.49 | 0.53 | 0.57 | 0.60 | 0.64 |
| 14.2 | 0.42 | 0.45 | 0.49 | 0.53 | 0.57 | 0.60 | 0.64 |
| 14.4 | 0.42 | 0.45 | 0.49 | 0.53 | 0.57 | 0.60 | 0.64 |
| 14.6 | 0.42 | 0.45 | 0.49 | 0.53 | 0.56 | 0.60 | 0.63 |
| 14.8 | 0.42 | 0.45 | 0.49 | 0.53 | 0.56 | 0.60 | 0.63 |
| 15 | 0.42 | 0.45 | 0.49 | 0.52 | 0.56 | 0.60 | 0.63 |
| 15.2 | 0.41 | 0.45 | 0.49 | 0.52 | 0.56 | 0.60 | 0.63 |
| 15.4 | 0.41 | 0.45 | 0.48 | 0.52 | 0.56 | 0.60 | 0.63 |
| 15.6 | 0.41 | 0.45 | 0.48 | 0.52 | 0.56 | 0.60 | 0.63 |
| 15.8 | 0.41 | 0.45 | 0.48 | 0.52 | 0.56 | 0.60 | 0.63 |
| 16 | 0.41 | 0.45 | 0.48 | 0.52 | 0.56 | 0.60 | 0.63 |
| 16.2 | 0.41 | 0.45 | 0.48 | 0.52 | 0.56 | 0.59 | 0.63 |
| 16.4 | 0.41 | 0.45 | 0.48 | 0.52 | 0.56 | 0.59 | 0.63 |
| 16.6 | 0.41 | 0.45 | 0.48 | 0.52 | 0.56 | 0.59 | 0.63 |
| 16.8 | 0.41 | 0.45 | 0.48 | 0.52 | 0.56 | 0.59 | 0.63 |
| 17 | 0.41 | 0.45 | 0.48 | 0.52 | 0.56 | 0.59 | 0.63 |
| 17.2 | 0.41 | 0.44 | 0.48 | 0.52 | 0.56 | 0.59 | 0.63 |
| 17.4 | 0.41 | 0.44 | 0.48 | 0.52 | 0.56 | 0.59 | 0.62 |
| 17.6 | 0.41 | 0.44 | 0.48 | 0.52 | 0.56 | 0.59 | 0.62 |
| 17.8 | 0.41 | 0.44 | 0.48 | 0.52 | 0.55 | 0.59 | 0.62 |
| 18 | 0.41 | 0.44 | 0.48 | 0.52 | 0.55 | 0.59 | 0.62 |
